# A randomized phase II study of SM‐88 plus methoxsalen, phenytoin, and sirolimus in patients with metastatic pancreatic cancer treated in the second line and beyond

**DOI:** 10.1002/cam4.4768

**Published:** 2022-05-02

**Authors:** Marcus S. Noel, Semmie Kim, Marion L. Hartley, Steve Wong, Vincent J. Picozzi, Harry Staszewski, Dae Won Kim, Jan M. Van Tornout, Philip Agop Philip, Vincent Chung, Allyson J. Ocean, Andrea Wang‐Gillam

**Affiliations:** ^1^ Georgetown Lombardi Comprehensive Cancer Center Washington District of Columbia USA; ^2^ TYME Technologies Inc. Bedminster New Jersey USA; ^3^ The Ruesch Center for the Cure of Gastrointestinal Cancers Washington District of Columbia USA; ^4^ Sarcoma Oncology Research Center Santa Monica California USA; ^5^ Virginia Mason Hospital and Medical Center Seattle Washington USA; ^6^ Mather Hospital Port Jefferson New York USA; ^7^ The University of Texas MD Anderson Cancer Center Houston Texas USA; ^8^ Karmanos Cancer Center Wayne State University Michigan Detroit USA; ^9^ SWOG Farmington Hills Michigan USA; ^10^ City of Hope Duarte California USA; ^11^ Weill Cornell Medicine New York‐Presbyterian Hospital New York New York USA; ^12^ Washington University School of Medicine in St. Louis St. Louis Missouri USA

## Abstract

**Background:**

This trial explores SM‐88 used with methoxsalen, phenytoin, and sirolimus (MPS) in pretreated metastatic pancreatic ductal adenocarcinoma (mPDAC)

**Methods:**

Forty‐nine patients were randomized to daily 460 or 920 mg oral SM‐88 with MPS (SM‐88 Regimen). The primary endpoint was objective response rate (RECIST 1.1).

**Results:**

Thirty‐seven patients completed ≥ one cycle of SM‐88 Regimen (response evaluable population). Disease control rate (DCR), overall survival (OS), and progression‐free survival (PFS) did not differ significantly between dose levels. Stable disease was achieved in 9/37 patients (DCR, 24.3%); there were no complete or partial responses. Quality‐of‐life (QOL) was maintained and trended in favor of 920 mg. SM‐88 Regimen was well tolerated; a single patient (1/49) had related grade 3 and 4 adverse events, which later resolved. In the intention‐to‐treat population of 49 patients, the median overall survival (mOS) was 3.4 months (95% CI: 2.7–4.9 months). Those treated in the second line had an mOS of 8.1 months and a median PFS of 3.8 months. Survival was higher for patients with stable versus progressive disease (any line; mOS: 10.6 months vs. 3.9 months; *p* = 0.01).

**Conclusions:**

SM‐88 Regimen has a favorable safety profile with encouraging QOL effects, disease control, and survival trends. This regimen should be explored in the second‐line treatment of patients with mPDAC.

ClinicalTrials.gov Identifier: NCT03512756.

## Graphical abstract

This trial explores 460 or 920 mg oral SM‐88 daily, used with methoxsalen, phenytoin, and sirolimus (MPS) in previously treated patients with metastatic pancreatic ductal adenocarcinoma (mPDAC). SM‐88 + MPS was well tolerated (1 of 49 patients had drug‐related grade 3/4 adverse events), patient quality‐of‐life was maintained, and overall survival trended toward being better for patients treated in the second (median overall survival [mOS], 8.1 months; median progression‐free survival [mPFS], 3.8 months) versus ≥ third line (mOS, 3.7 months; mPFS, 1.8 months). SM‐88 + MPS should be further explored in the second‐line treatment of patients with mPDAC.

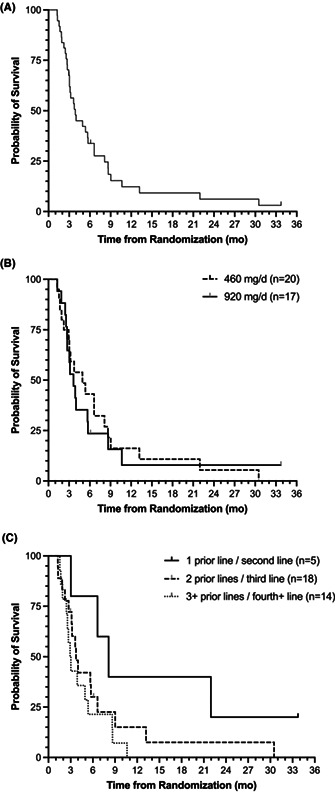

## 1 BACKGROUND

Annually, approximately 60,000 individuals living in the United States^1^, ^2^, ^3^ and almost half a million people globally^1^ are diagnosed with pancreatic cancer. In 2020, 47,050 people in the United States^4^ and 466,003 people globally^1^ died from this disease. Worldwide, both incidence and mortality rates due to pancreatic cancer have been increasing over time. In Europe, it is predicted that by 2025, pancreatic cancer may surpass breast cancer as the third leading cause of cancer death,^1^ and in the United States, by 2030, pancreatic cancer will be the second leading cause of cancer mortality.^5^

Cytotoxic chemotherapy for pancreatic cancer is marginally effective due to a number of disease characteristics, which include the existence of a dense stromal network that inhibits drug delivery, an immunosuppressive microenvironment that enhances rapid tumor growth, and an altered cellular metabolism compared to normal cells that can render patients nutritionally deplete.^6^

Presently, the first‐line treatment options for patients with metastatic pancreatic ductal adenocarcinoma (mPDAC) are FOLFIRINOX, which is associated with a median overall survival (mOS) of 11.7 months, or gemcitabine plus nab‐paclitaxel, which is associated with an mOS of 8.7 months.^7^, ^8^ However, the survival advantage from FOLFIRINOX often comes at the cost of severe toxicity. Subsequent second‐line FDA‐approved therapy is nanoliposomal irinotecan (nal‐IRI) combined with 5‐fluorouracil (5‐FU) plus leucovorin (LV), which provides a further mOS of 6.1 months compared to only 4.2 months for 5‐FU/LV alone.^9^ There are no FDA‐approved therapies in the third line and beyond for patients with mPDAC.

Preclinical studies have affirmed the role of oxidative stress in carcinogenesis.^10^ Cellular oxidative stress occurs due to reactive oxygen and nitrogen species, generated as byproducts of normal cell metabolism, immune response, and inflammation.^11^ Reactive oxygen and nitrogen species interact with and damage DNA, producing mutations and genomic instability (a precancerous state) and eventually malignant transformations (a cancerous state).^12^ Yet, these changes are also proposed as a therapeutic mechanism for targeted cell death.^13^, ^14^

Oral SM‐88 (racemetyrosine; D,L‐alpha‐metyrosine) is a dysfunctional derivative of tyrosine intended to be non‐functional for protein synthesis and comprises an equal proportion of the D‐ and L‐ stereoisomers of alpha metyrosine. It is administered with low oral doses (smaller than those used for approved indications) of three repurposed agents: methoxsalen, 10 mg; phenytoin, 50 mg; and sirolimus, 0.5 mg (MPS). Human cancers have increased uptake of tyrosine‐analogs relative to normal cells.^15^ Once inside the cancer cell, the SM‐88 regimen leverages the known hypoxic and anaerobic metabolism of cancer (also known as the Warburg effect) to enhance and target the delivery of its non‐functional amino acid and generated reactive lipid species.^16^, ^17^, ^18^, ^19^, ^20^, ^21^, ^22^ It is hypothesized that the denatured tyrosine then disrupts cancer cell regulatory protein synthesis, weakening cancer cell defenses. The repurposed agents are used to (1) increase absorption of the altered tyrosine into the tumor environment through increased cellular ketosis (sirolimus), (2) increase the production of reactive lipid species (phenytoin), and (3) enhance the effect of oxidative stress inside the cancer cell (methoxsalen). The SM‐88 regimen leads to apoptosis by augmenting tumor intracellular oxidative stress and removing critical mechanisms to regulate ROS.^15^, ^16^

SM‐88 as a novel amino acid derivative (D,L‐alpha‐metyrosine; racemetyrosine) used in combination with melanin, Melanotan II, phenytoin, and sirolimus (SMK Therapy), delivered in multiple dosage forms, was previously evaluated in a first‐in‐human (FIH) study of 30 patients with a range of solid tumors. This combination was safe and well tolerated; all treatment‐related adverse events were grade 1 or 2, with hyperpigmentation and fatigue being most frequent.^23^ Outcomes of 12 patients with mPDAC—3 treated with SMK Therapy as part of the FIH study^23^ and nine compassionately treated with SMK Therapy—were presented at the 2018 ASCO Gastrointestinal Cancers Symposium.^24^ All 12 patients had progressive disease upon entry into the analysis and received a variety of prior treatment regimens (median one prior line of systemic therapy with a range of 0–6 prior lines). Ten of these 12 patients were administered at least one 6‐week cycle of SMK Therapy and were evaluable for response. Seven of these 10 response‐evaluable patients received SMK Therapy alone; 3/10 received SMK Therapy plus 5‐FU‐based chemotherapy. All seven evaluable patients treated with SMK Therapy alone maintained or improved ECOG performance status while on treatment and did not experience experimental drug‐related SAEs. Three of these 7 (43%) patients had >12 months OS, 1/7 had a CR, and 1/7 had a PR. The seven‐patient median progression‐free survival (mPFS) was 4.0 months.^24^

SM‐88 at doses of up to 460 mg/day, used with MPS, was not associated with any dose‐limiting toxicity (DLT).^25^, ^26^ Here, we describe a study of oral SM‐88 with MPS in patients with PDAC previously treated with at least one line of chemotherapy. We compared response, survival, and adverse event (AE) data for patients treated with two different SM‐88 doses (460 and 920 mg/day).

## 2 METHODS

### 2.1 Patients

Patients ≥18 years of age with histologically confirmed PDAC were eligible for this study if they also had evidence of measurable metastatic disease using response evaluation criteria in solid tumors (RECIST) version 1.1 and had progressed on one or more systemic therapies. Subjects were required to have an ECOG performance status of ≤2. Patients were also required to have adequate organ function, defined as follows: platelets ≥100 × 10^9^/L; ANC ≥1.5 × 10^9^/L; AST/ALT ≤2.5 × upper limit of normal (ULN); total or conjugated bilirubin ≤1.5 × ULN; and serum creatinine ≤1.5 × ULN or creatinine clearance (CrCl) ≥60 ml/min as calculated by the Cockroft–Gault method. Patients were also required to have completed any investigational treatment at least 30 days before the first dose of SM‐88 plus MPS.

### 2.2 SM‐88

Oral SM‐88 was given at doses of 460 and 920 mg daily, divided in a BID administration, together with fixed once‐daily oral dosing of MPS (methoxsalen, 10 mg; phenytoin, 50 mg; sirolimus, 0.5 mg). All dosing was daily and continuous, administered in consecutive 28‐day cycles. Hereafter, SM‐88 with MPS will be termed “SM‐88 Regimen.”

### 2.3 Study design

In this phase II study, patients were randomized 1:1 to 460 or 920 mg/day SM‐88 with MPS using a block randomization scheme. No stratification factors were used. Treatment was continued until disease progression (as assessed by study investigators) or unacceptable toxicity. Subsequent therapies were administered at the treating physician's discretion. Patients were followed up in the clinic 28 days after treatment cessation and then at 3‐month intervals via phone or in‐person for survival data. On signs of radiologic progression on SM‐88 Regimen, the investigator and patient could petition to continue treatment until progression was confirmed on subsequent imaging analysis, as long as there was a clinical benefit and no other therapeutic intervention was available. The study's primary endpoint was objective response rate (ORR; CR + PR) as defined by modified RECIST version 1.1 under blinded independent central review. In this study, a single RECIST‐based diagnosis of progression of disease was considered a validated response. Secondary endpoints included OS and PFS, defined as the time from the first dose administration until disease progression or death by any cause.

### 2.4 Efficacy and safety assessments

All subjects receiving at least one 28‐day cycle of the study drug (minimum 23 days on treatment for one cycle) or at least 50% of the prescribed doses over the first 8 weeks of the trial were included in efficacy evaluations provided that a baseline assessment and at least one post‐baseline evaluation were available, that is, the evaluable population (*n* = 37).

All randomized subjects were included in the compilation of baseline and demographic characteristics, that is, the intention to treat (ITT) population (*n* = 49). All subjects receiving at least one dose of SM‐88 Regimen were included in the safety analyses, that is, the safety population (*n* = 48).

Secondary endpoints included OS and PFS, defined as the time from randomization until disease progression or death by any cause; recommended Phase 2 dose; and quality‐of‐life (QOL).

Tumor response was evaluated by computed tomography (CT) or, for those with contraindications to CT, by magnetic resonance imaging according to RECIST v. 1.1.^27^ The response was assessed at baseline (at most, 14 days before the first dose of SM‐88 Regimen) and then every 8 weeks (on Day 28 [± 5 days] of Cycles 2, 4, and 6) while patients were on study therapy.

AEs were graded according to National Cancer Institute Common Terminology Criteria for Adverse Events (NCI CTCAE) version 4.03.^28^

### 2.5 Quality‐of‐life

Health‐related QOL was assessed using the 30‐item European Organization for Research and Treatment of Cancer Quality‐of‐Life Questionnaire (EORTC QLQ‐C30) and a pancreatic‐specific module. The patients completed these questionnaires at baseline, every 4 weeks (day 1 of every cycle) until disease progression, and at discontinuation of the trial intervention, within the 28 days following the last dose of the trial agent. Global scores on the EORTC QLQ‐C30 range from 0 to 100, with higher scores indicating better QOL; an increase or decrease of at least 10 points was considered a clinically meaningful change.^21^

### 2.6 Statistical analysis

The null hypothesis (H‐0) was that an ORR of <8% (half that found for the NAPOLI‐1 trial) made SM‐88 Regimen an unattractive option. The alternative hypothesis (H‐A) used to power the study was ≥8%. Survival data were collected for all randomized patients (all patients in our ITT population). Safety data were summarized in patients who received at least one dose of a trial agent (the safety population). Overall outcomes were examined by SM‐88 dose. Health‐related QOL was assessed in all patients for whom an EORTC QLQ‐C30 was available and evaluable at baseline. The distribution of scores and changes in scores over time for each question of the EORTC QLQ‐C30 and EORTC PAN‐26 were analyzed and stratified by the SM‐88 dose to which subjects were randomized. Scores at pre‐and post‐treatment time points were examined using box and whisker plots to compare median scores by group.

SAS® version 9.4 (SAS Institute Inc., Cary, NC) was used for statistical analyses. PFS and OS rates were estimated using the Kaplan–Meier method, and median survival times and their 95% confidence intervals were reported. The log‐rank test was used to assess and compare survival differences between the groups. A *p* value of <0.05 was considered statistically significant. A Cox proportional‐hazards analysis was performed to estimate the hazard ratio (HR) for OS between subjects who reached stable disease versus subjects with progressive disease.

This study received approval from institutional IRBs, and all subjects provided written informed consent to participate. In addition, the study was performed in accordance with the Declaration of Helsinki.

## 3 RESULTS

### 3.1 Patient characteristics

A total of 99 patients with metastatic PDAC were screened, and of those, 49 patients (ITT population) were enrolled between May 1, 2018, and March 12, 2019. Thirty‐seven of the 49 patients completed at least one 28‐day cycle of therapy (i.e., 28+ days, *n* = 31; 27 days, *n* = 3; 26 days, *n* = 2; 23 days, *n* = 1) and were evaluable for response (evaluable population). Demographics and baseline characteristics were similar between the ITT and evaluable groups (Table 1). Overall, the median age of the patient population was 66.4 years, 51% of subjects were male, and 90% were white (Table 1). The patient population was heavily pretreated; more than 85% had received at least two previous lines of treatment. All had progressive disease on their last treatment.

**TABLE 1:** Patient demographics

Demographics	ITT, *n* = 49	Evaluable, *n* = 37
Age, years ± SD	66.9 ± 10.4	66.9 ± 10.6
Gender, female, *n* (%)	24 (49.0%)	17 (45.9%)
ECOG performance status at screening		
Score of 0, *n* (%)	15 (30.6%)	12 (32.4%)
Score of 1, *n* (%)	33 (67.4%)	25 (67.6%)
Score of 2, *n* (%)	1 (2.0%)	0 (0.0%)
Body mass index ± SD	23.6 ± 4.4	23.5 ± 4.3
Race, *n* (%)		
White	44 (89.8%)	34 (91.9%)
Black or African American	3 (6.1%)	2 (5.4%)
Asian	2 (4.1%)	1 (2.7%)
Prior radiotherapy, *n* (%)	15 (30.6%)	12 (32.4%)
Prior surgery, *n* (%)	19 (38.8%)	16 (43.2%)
Prior lines of therapy, *n* (%)		
1	7 (14.3%)	5 (13.5%)
2	24 (49.0%)	18 (48.6%)
3	10 (20.4%)	9 (24.3%)
4+	8 (16.3%)	5 (13.5%)
Prior systemic therapy, *n* (%)^a^		
Gemcitabine	42 (85.7%)	32 (86.5%)
5‐fluorouracil	40 (81.6%)	31 (83.8%)
Irinotecan	37 (75.5%)	28 (75.7%)
Platinum	36 (73.5%)	28 (75.7%)
FOLFIRINOX or mFOLFIRINOX	32 (65.3%)	23 (62.2%)
FOLFOX or OFF	1 (2.2%)	1 (2.7%)
FOLFIRI or IFL	9 (18.4%)	7 (18.9%)
Taxanes	35 (71.4%)	27 (73.0%)
Nab‐paclitaxel	34 (69.4%)	26 (70.3%)
Docetaxel	1 (2.0%)	1 (2.7%)
PARP inhibitors	2 (4.1%)	1 (2.7%)
Immunotherapy	6 (12.2%)	3 (8.1%)
Other investigational agents	9 (18.4%)	7 (18.9%)

Abbreviation: ITT, intent‐to‐treat.

^a^ Indicates exposure to drug alone, or as part of any regimen.

### 3.2 Antitumor response

Of the 37 evaluable patients, none showed an objective response (i.e., CR or PR) to therapy, although nine patients (24.3%; seven females and two males) had a best response of stable disease (SD by RECIST). Three of 37 patients (8.1%) had two consecutive scans showing SD by RECIST. Twenty‐two patients (59.5%) had progressive disease. Six subjects (16.2%) discontinued study participation before their first follow‐up scan on treatment could be completed and, therefore, did not have any RECIST responses available. Three out of five (60%) patients who received only one prior line of therapy had SD compared to 4/18 (22.2%) for two lines and 1/9 (11.1%) for three lines. Twelve subjects randomized to treatment (and included in the ITT group) were excluded from the evaluable group because they did not complete one full cycle of treatment. Reasons for this incomplete cycle include patient death (any cause), disease progression, or withdrawal of consent. Two of these 12 patients were treated in the second line, six in the third line, and four beyond the third line.

### 3.3 Quality of life

**FIGURE 1:**
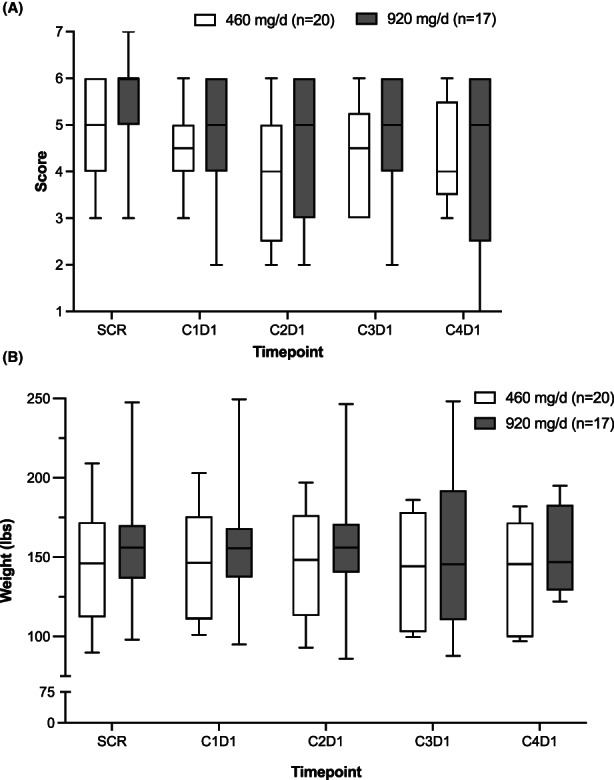
Patient overall quality‐of‐life scores on treatment, as measured by the EORTC QLQ‐C30 Question 30, and weight by the dose of SM‐88 Regimen. Box and whisker plots showing median, interquartile range, and minimum and maximum values of (A) EORTC QLQ‐C30 Question 30, “How would you rate your overall QOL during the past week?” from pre‐treatment to Cycle 4 on treatment; and (B) weight in pounds pre‐ and post‐treatment by dose group. In (A), scoring ranges from 1 to 7, where 1 = very poor and 7 = excellent. EORTC QLQ‐C30, European Organization for Research and Treatment of Cancer Quality‐of‐Life Questionnaire; QOL, quality‐of‐life

On entry into the study, all patients were required to have an ECOG performance status of ≤2. Subjects generally maintained weight during SM‐88 Regimen therapy, and six patients had a net gain in absolute weight from screening to Cycle 3 on treatment; 3 of these subjects had RECIST‐verified stable disease. Patients also maintained QOL and their global EORTC questionnaire health scores throughout their treatment with the study drug. It appeared that patients receiving 920 mg/day SM‐88 Regimen had slightly improved overall health and QOL scores (Figure 1A) compared to patients receiving the 460 mg dose, particularly for the first four cycles for which sufficient data were collected. However, this difference did not reach statistical significance.

### 3.4 Adverse events

All 49 patients in the ITT population were followed for toxicity and tolerability; 48 of these subjects reported at least one AE (all‐cause, any grade; 26 in the low‐dose group and 22 in the high‐ dose group). SM‐88 Regimen had a favorable safety profile. None of the Grade 1 or 2 events observed were determined to be definitively *related* to SM‐88 Regimen. Grade 1 or 2 events deemed *probably related* to SM‐88 Regimen were fatigue (*n* = 3) and nausea (*n* = 2).

Regarding treatment‐emergent serious (Grade 3/4) AEs, among the safety population (*n* = 48), 27 distinct subjects experienced a Grade 3 or 4 event (25 experienced any Grade 3 event; 7 experienced any Grade 4 event). Two patients had one documented Grade 5 event each; hence, one patient had intractable nausea but died from progressive disease, and one patient had spontaneous bacterial peritonitis, which was listed as the cause of death. Both events were determined to be *unrelated* to the study treatment.

There were only three *related* serious AEs (Grade 3 or 4), all of which were reported in the same single subject. This patient received 920 mg/day SM‐88 Regimen. The first event was “abdominal pain” (Grade 3/severe), and the second and third events were “hypotension” (both Grade 4/life threatening). All three events were documented as having an outcome of “recovered/resolved.” SM‐88 Regimen was held until AE resolution, and then treatment was resumed and continued for another two cycles (8 weeks).

Two subjects receiving 460 mg/day SM‐88 Regimen were documented as having pulmonary embolism: one Grade 3 and one Grade 4 event. However, both were deemed *unrelated* to SM‐88 Regimen. In an additional subject receiving 460 mg/day, Grade 3 deep vein thrombosis (brachial vein) was recorded but again deemed *unrelated* to SM‐88 Regimen. Another patient, this time receiving 920 mg/day SM‐88 Regimen, had a documented Grade 3 thromboembolic event, also judged *unrelated* to SM‐88, which was resolved. One event of portal vein thrombosis (Grade 4) was documented in one more subject in the 920 mg/day arm and deemed possibly related, with eventual recovery/resolution. Two subjects (one in each dose group) reported a pleural effusion. Both events were Grade 3, *unrelated* to study treatment, and had an outcome of recovered/resolved (Table 2).

**TABLE 2:** Treatment‐emergent serious adverse events. Treatment‐emergent serious adverse events (Grades 3 and 4) reported among treated subjects (safety population, *n* = 48) with event frequency >1, displayed by SM‐88 dose. Among all documented SAEs, there were no (*n* = 0) suspected unexpected serious adverse reactions

	460 mg/day (*N* = 25)		920 mg/day (*N* = 23)	
	*n*	%	*n*	%
Any Grade 3 or 4 event	14	56.0	13	56.5
Grade 3	11	44.0	9	39.1
Grade 4	3^a^	12.0	4^a^	17.4

	Grade 3		Grade 4		Grade 3		Grade 4	
	*n*	%	*n*	%	*n*	%	*n*	%
Abdominal pain	1	4.0	0	0.0	6	26.1	0	0.0
Anemia	2	8.0	0	0.0	3	13.0	0	0.0
Thromboembolic event^b^	2	8.0	1	4.0	1	4.3	1	4.3
Ascites	1	4.0	0	0.0	1	4.3	0	0.0
Increased bilirubin	1	4.0	1	4.0	0	0.0	0	0.0
Sepsis	0	0.0	0	0.0	0	0.0	2	8.7
Cholangitis	0	0.0	0	0.0	1	4.3	1	4.3
Hyponatremia	1	4.0	0	0.0	2	8.7	0	0.0
Pleural effusion	1	4.0	0	0.0	1	4.3	0	0.0
Biliary obstruction	1	4.0	0	0.0	1	4.3	0	0.0

*Note*: In this table, only abdominal pain (Grade 3) and hypotension (Grade 4) in a single patient were deemed related to SM‐88 Regimen.

Abbreviation: *n*, number of subjects.

^a^ Subjects who reported both Grade 3 and 4 events are included only in the Grade 4 row.

^b^ Thromboembolic events included the following: pulmonary embolism (*n* = 2); deep vein thrombosis (*n* = 1); portal vein thrombosis (*n* = 1); thromboembolic event, not otherwise specified (*n* = 1).

### 3.5 Patient survival

**FIGURE 2:**
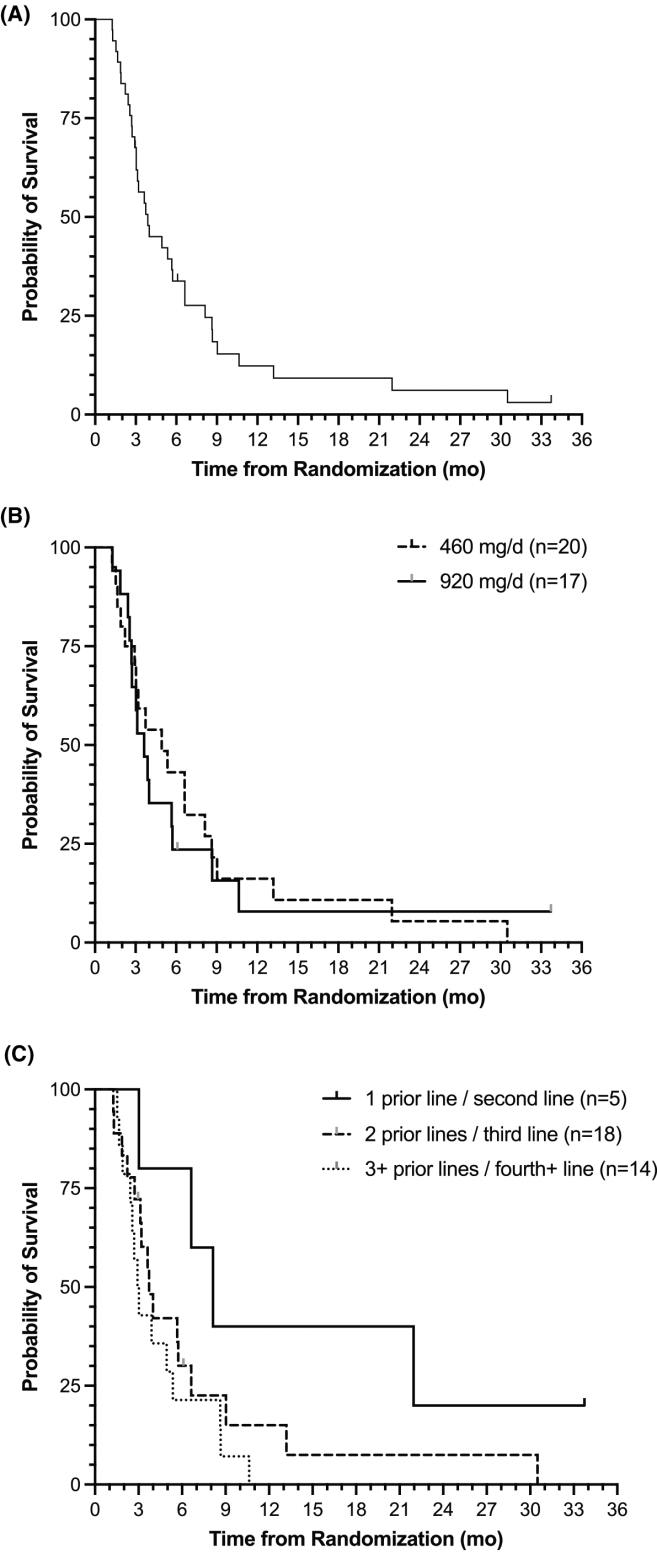
Overall survival (OS) of evaluable patients receiving SM‐88 Regimen (*n* = 37). Simple Kaplan– Meier curve of OS in months for (A) all 37 patients (median 3.9 months; 95% CI: 3.0–5.7), (B) the 20 patients receiving 460 mg/day compared to the 17 patients receiving 920 mg/day (4.9 months [95% CI: 2.2–8.1] vs. 3.6 months [95% CI: 2.6–5.7]; *p* = 0.79) and (C) patients receiving SM‐88 Regimen after 1 prior line (*n* = 5), 2 prior lines (*n* = 18), and 3 or more prior lines (*n* = 14) of therapy: 8.1 months (95% CI: 3.0 ‐ n/a [not reached]) vs. 3.7 months (95% CI: 2.7–6.6) versus 3.0 months (95% CI: 1.9–5.4); log‐rank *p* = 0.08

In the ITT population of 49 patients, the mOS was 3.4 months (95% CI: 2.7–4.9 months). For the 37 patients evaluable for response, receiving both doses (460 and 920 mg/day) of SM‐88 Regimen, the mOS was 3.9 months (95% CI: 3.0–5.7) (Figure 2). There was no significant difference in OS between the two doses by log‐rank test (460 mg/day [*n* = 20], 4.9 months [95% CI: 2.2–8.1]; 920 mg/day [*n* = 17], 3.6 months [95% CI: 2.6–5.7]; *p* = 0.79).

**FIGURE 3:**
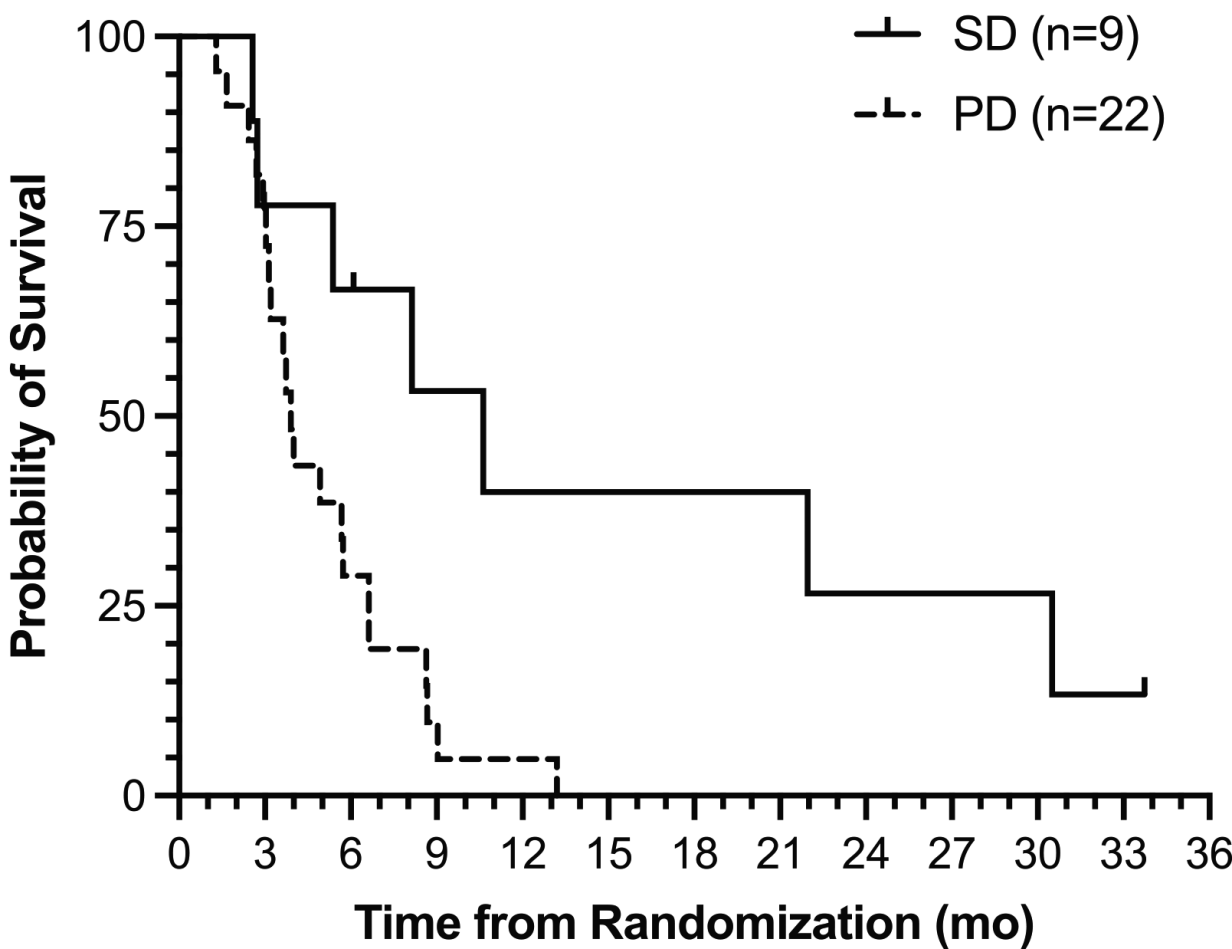
OS of evaluable patients by RECIST best response (SD or PD). Kaplan–Meier OS curve for evaluable patients receiving SM‐88 Regimen who achieved stable disease (solid line; mOS = 10.6 months [95% CI: 2.6–30.5]) versus those who had disease progression (hashed line; mOS = 3.9 months [95% CI: 3.0–5.7]). Log‐rank *p* = 0.01; HR = 0.29 (95% CI: 0.10–0.79). mOS, median overall survival; OS, overall survival; RECIST, response evaluation criteria in solid tumors; SD, stable disease

The OS for patients who achieved stable disease (10.6 months [95% CI: 2.6–30.5]) was significantly higher than those with progressive disease on treatment (3.9 months [95% CI: 3.0–5.7]; log‐rank *p* = 0.01; HR = 0.29 [95% CI: 0.10–0.79]; Figure 3).

When breaking down survival in the evaluable population by number of prior lines of therapy, there was a trend toward longer patient OS with fewer lines of prior therapy (1 prior line [*n* = 5], 8.1 months [95% CI: 3.0 ‐ no upper limit]; 2 prior lines [*n* = 18], 3.7 months [95% CI: 2.7–6.6]; 3 or more prior lines [*n* = 14], 3.0 months [95% CI: 1.9–5.4]; log‐rank *p* = 0.08) (Figure 2C). When separating patients by a prior line of therapy *and* dose received, there were not enough patients in each group to make a statistical comparison (data not shown).

The mPFS for the ITT population was 1.8 months (95% CI: 1.7–2.0 months). The mPFS of the evaluable population was 1.9 months (95% CI: 1.7–2.0). There were no significant differences according to dose (mPFS: 1.9 vs. 1.8 [95% CI: 1.6–3.0 vs. 1.0–2.6] for 460 vs. 920 mg/day, respectively; log‐rank *p* = 0.25).

**FIGURE 4:**
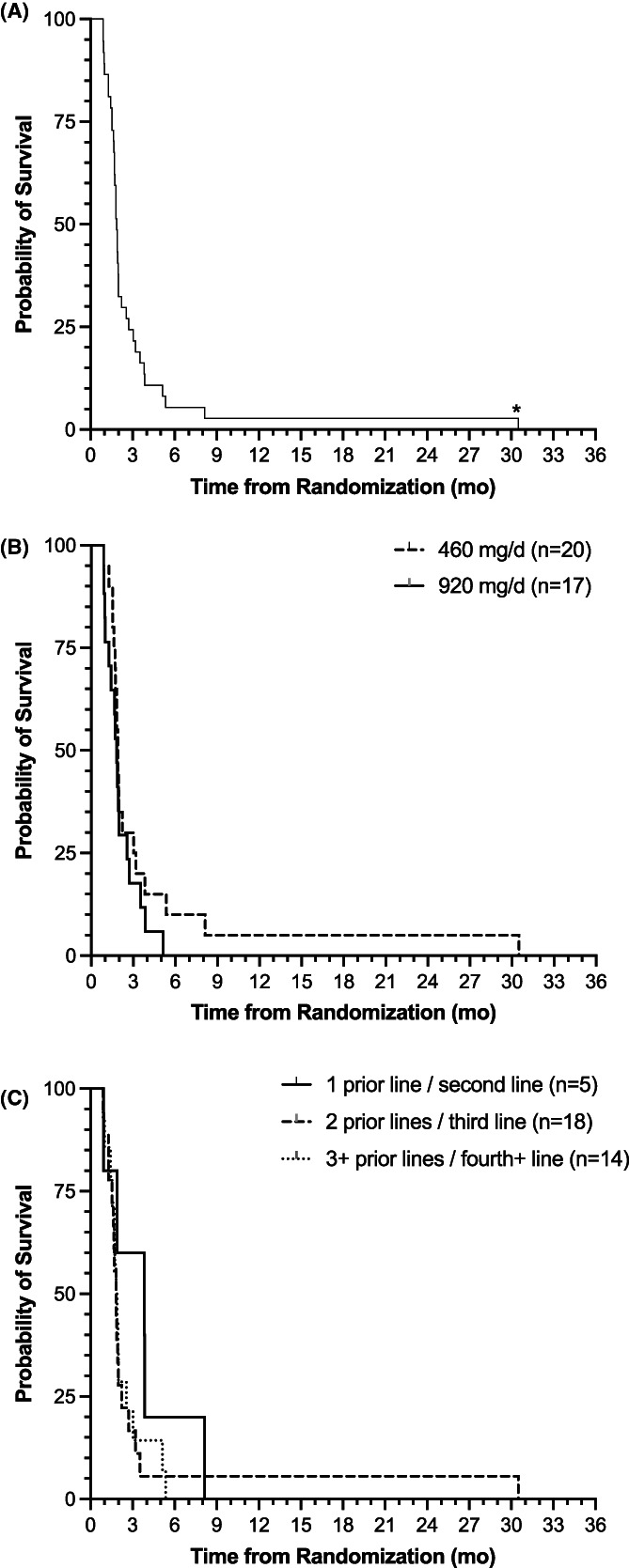
PFS curves for all evaluable patients (*n* = 37). Kaplan–Meier curve of PFS in months for (A) all 37 evaluable patients (median 1.9 months [95% CI: 1.7‐2.0]), (B) the 20 patients receiving 460 mg/day (1.9 months [95% CI: 1.6–3.0]) compared to the 17 patients receiving 920 mg/day (1.8 months [95% CI: 1.0–2.6]; log‐rank *p* = 0.25), and (C) patients receiving SM‐88 Regimen after 1 prior line (solid line; *n* = 5), 2 prior lines (black hashed line; *n* = 18), and 3 or more prior lines (black dotted line; *n* = 14) of therapy (3.8 months [95% CI: 0.9 – not reached], 1.8 months [95% CI: 1.5–2.0], and 1.9 months [95% CI: 1.4–2.6]; log‐rank *p* = 0.44). *A single patient who had received 2 lines of prior treatment showed a prolonged PFS response, with RECIST‐confirmed stable disease at 15 weeks post‐treatment initiation. PFS, progression‐free survival; RECIST, response evaluation criteria in solid tumors

There was a trend toward greater patient PFS with fewer prior lines of therapy: 1 prior line (*n* = 5), 3.8 months (95% CI: 0.9 – not reached); 2 prior lines (*n* = 18), 1.8 months (95% CI: 1.5–2.0); 3 or more prior lines (*n* = 14), 1.9 months (95% CI: 1.4–2.6; log‐rank *p* = 0.44; Figure 4).

## 4 DISCUSSION

Advanced PDAC does not respond well to traditional cytotoxic chemotherapy; approved regimens are associated with significant toxicity yet provide a 5‐year survival rate of only 3%.^29^ Based on favorable results from the NAPOLI‐1 trial, nal‐IRI/5‐FU/LV is approved in the second line for PDAC following disease progression on gemcitabine‐based therapy.^9^ In our study of SM‐88 Regimen, we hypothesized that an ORR of 8%, which approximates half the rate of the second‐line NAPOLI‐1 trial,^9^ would be clinically meaningful in our patient population comprising 13.5% second line and 86.5% third line and beyond.

Our study failed to meet its primary objective, yet we did see a DCR of 24.3% (any SM‐88 dose, any line). This DCR comprised SD only, but it was clinically meaningful in this highly refractory patient population. Certainly, in the FIH study of SM‐88 in patients with metastatic solid tumors, clinical benefits, defined as responses that were maintained on more than one follow‐up CT scan, were observed in subjects achieving a CR, PR, and also SD.^23^

Five of our patients were being treated in the second line, and three of these had stable disease while two had disease progression, making a DCR of 60% (60% SD). Although these patient numbers are very small and cannot be directly compared to larger trials, it should be noted that our second‐line (60%) and, in some cases, all‐line (24.3%) DCRs are characteristic of other studies, but without PRs and with considerably less toxicity. Our prior FIH^23^ and compassionate use^24^ experience of SM‐88 (racemetyrosine) in metastatic PDAC suggests a role for SM‐88 in advanced mPDAC. Given the fact that current second‐line treatments for patients with mPDAC remain suboptimal, we felt it was appropriate to offer SM‐88 Regimen to this group of individuals in addition to those being treated in later line settings.

Second‐line studies of more traditional chemotherapeutic regimens^9^, ^30^, ^31^, ^32^, ^33^, ^34^, ^35^, ^36^ found DCRs ranging from 13% for oxaliplatin/capecitabine^31^ to 58% for FOLFOX6.^32^ No CRs were found in any study, but a few studies found PRs by RECIST, ranging from 0% for mFOLFIRI3 (23% DCR)^33^ to 17% for nal‐IRI/5‐FU/LV (49% DCR).^9^, ^34^ A Phase II study of eryaspase plus chemotherapy produced a DCR of 48.4% (12.6% PR),^37^ yet this regimen recently failed to reach its primary endpoint in a Phase III study.^38^ In second‐line studies of targeted and immune therapy and their combinations, again, no CRs were observed, but there was a range of DCRs with PRs.^39^, ^40^, ^41^, ^42^ For example, erlotinib plus gemcitabine produced a DCR of 32% (0% PR),^39^ the CXCR4 antagonist BL‐8040 (motixafortide) in combination with pembrolizumab achieved a DCR of 34.5% (3.5% PR),^41^ and durvalumab plus tremelimumab, 9.4% (3.1% PR).^42^ Many studies do not report on confirmation of PRs, which could bring the reliability of their results into question. For example, investigators of durvalumab plus tremelimumab found that only one (3.1%) of their original three PRs could be confirmed.^42^

The minimal Grade 3/4 toxicity and lack of necessity for dose reductions seen with SM‐88 Regimen can be contrasted with the more severe AE profiles of the traditional chemotherapeutic and targeted agents and immunotherapies listed above, all of which required dose reductions and/or cessation due to toxicity.^30^, ^31^, ^32^, ^33^, ^35^, ^37^, ^39^, ^40^, ^41^, ^42^ Even the approved nal‐IRI/5‐FU/LV regimen was associated with appreciable Grade 3/4 toxicity (27% neutropenia, 13% diarrhea, 11% vomiting, and 14% fatigue), and 33% of patients had to undergo dose reductions.^9^, ^34^

In our study, 32 patients (86.5%) were treated in the third line and beyond. These patients had a DCR of 18.8% (six out of 32 patients had SD). This DCR, in context, is similar to that achieved in other third‐line studies, although prospective data are limited: a Phase IIb trial of GVAX pancreas plus CRS‐207 plus cyclophosphamide found a DCR of 23.5%,^43^ a Phase II study of ganetespib found a DCR of 21.4%,^44^ and a Phase I trial of ribociclib plus everolimus, 18%.^45^ A Phase 1b trial of ^90^Y‐clivatuzumab tetraxetan plus gemcitabine found a DCR of 41%,^46^ but a subsequent Phase III trial (PANCRIT‐1; NCT01956812) was closed due to a high patient death rate.^47^ SM‐88 Regimen in the third line was more favorable than all other regimens with respect to toxicity.

In previous trials of SM‐88, no serious drug‐related AEs were reported (Grade ≥3),^23^ and to date, including in this study, SM‐88 Regimen has not been associated with DLT.^25^, ^26^ In this trial, only 2% grade 3/4 treatment‐related AEs were observed, which occurred at the 920 mg/day dose. In one patient, SM‐88 was held until resolution of abdominal pain and hypotension before being continued at the same dose for more than two cycles. Of note, despite the interruption in treatment, this patient showed a good level of tumor shrinkage. No differences in toxicity were observed between the 460 and 920 mg/day SM‐88 Regimen dosing. Compared to other second and third‐line treatments, the toxicity of SM‐88 Regimen was mild, very manageable, and did not require dose reductions nor significant dose interruptions.

Patient QOL, assessed via EORTC QLQ‐C30, was maintained during this trial (Figure 1), which contrasts with the deterioration of QOL observed for patients treated with mFOLFOX6 and 5‐FU/LV in the second‐line PANCREOX trial.^32^ Of note, for patients in the first line, QOL can improve when treatment is effective. (e.g., FOLFIRINOX),^48^ but few studies tackle this issue in patients with refractory disease near the end of their lives. Prigerson et al.^49^ showed that in patients with progressive metastatic cancer, including PDAC, with an ECOG performance score of 1 at study entry, chemotherapy induced a worsening in QOL when given at the end of life (odds ratio [OR], 0.35; 95% CI, 0.17–0.75). This could indicate that chemotherapy given to patients with refractory cancer might reduce QOL when patients are near death. In our study, SM‐88 Regimen appeared to provide QOL benefit to many patients near end‐of‐life; 73.7% of our evaluable patients died before they could complete five cycles of SM‐88 Regimen. Still, patients maintained their overall QOL and weight scores up to cycle 4 (Figure 1), likely because of minimal side effects and possibly due to disease control for the 10 patients with SD. Moreover, SM‐88 Regimen is orally dosed and avoids infusion suite visits and their related aggravations; the value of this added benefit and freedom may not be fully appreciated by QOL scores.

Considering survival, in our evaluable patients (all lines of therapy; all responses), mOS was 3.9 months: 4.9 months for 460 mg/day and 3.6 months for the 920 mg/day SM‐88 Regimen (*p* = 0.79). However, patients with stable disease (any line or dose) appeared to have significantly longer mOS (10.6 months) than those with PD (3.9 months; *p* = 0.01). As anticipated, the mOS of the five patients treated in the second line was longer (8.1 months) than for patients treated in the third line (*n* = 18; 3.7 months) or beyond (*n* = 14; 3.0 months; *p* = 0.08). These few patients treated with SM‐88 Regimen in the second line had an mOS in keeping with the FDA‐approved nanoliposomal‐IRI (6.1 months^9^) and many other studied chemotherapeutic regimens in the second line; for example, 5‐FU/LV (3.3 months^50^ and 9.9 months^32^), FOLFOX (5.9 months^50^) and FOLFOX6 (6.1 months^32^), mFOLFIRI3 (5.3 months^33^), docetaxel plus capecitabine (6.3 months^35^), and gemcitabine plus nab‐paclitaxel (7.6 months).^36^ Although the Phase II study of eryaspase plus chemotherapy found an OS of 6 months,^37^ the Phase III investigation of eryaspase plus chemotherapy failed to show that the addition of eryaspase to chemotherapy improved OS in patients with advanced PDAC treated in the second line: mOS was 7.5 months versus 6.7 months with chemotherapy alone (HR, 0.92; 95% CI, 0.76–1.11; *p* = 0.375).^38^ Finally, to date, patient median OS following targeted and immune therapies has been mostly disappointing: gefitinib plus docetaxel, 4.5^40^; erlotinib/gemcitabine, 3.8^39^; ruxolitinib plus capecitabine^51^; and durvalumab plus tremelimumab, 3.1.^42^ The exception possibly lies with BL‐8040 plus pembrolizumab and chemotherapy, where the mOS was 3.3 months in the patient population previously treated with one line of therapy or more, but 7.5 months when taking those treated in the second line only.^41^

The mOS of patients treated with SM‐88 Regimen in the third line was 3.7 months, which is in line with other published trials. Hence, the trial of ribociclib plus everolimus found an mOS of 3.7 months^45^ the GVAX trial found an mOS between 3.7 and 5.4 months in their experimental arms versus 4.6 months in their chemotherapy arm^43^; and the Phase II ^90^Y‐clivatuzumab tetraxetan trial found an mOS of 7.9 months in patients receiving multiple cycles of this experimental therapy plus gemcitabine versus 3.4 months with chemotherapy alone.^46^ Regarding the latter trial, the closure of the subsequent Phase III trial (PANCRIT‐1; NCT01956812) possibly negates the Phase II findings.^47^ All experimental regimens were reported to have greater toxicity than SM‐88 Regimen.

With respect to mPFS, that obtained for second‐line SM‐88 Regimen was 3.8 months. In prospective trials of traditional chemotherapy, targeted therapy, and immunotherapy combinations, the mPFS in the second line ranged from 1.5 to 3.1 months.^9^, ^31^, ^32^, ^33^, ^35^, ^37^, ^40^, ^42^, ^50^ The mPFS achieved in the third line for SM‐88 Regimen was 1.8 months versus an equivalent 1.8 months for ribociclib plus everolimus,^45^ 2.3 months for GVAX, and 2.1 months for chemotherapy.^43^

We believe that our OS and PFS findings indicate a potential role for SM‐88 Regimen in the second‐line for patients with mPDAC, especially as the 920 mg dose appeared to be much more tolerable than traditional chemotherapy. In light of limited second‐line options for patients with mPDAC, further exploration of the benefit of the SM‐88 Regimen should be considered. Our results may support the possibility of combining SM‐88 with other established therapies, especially considering SM‐88 Regimen's apparent safety and its quite different mechanism of action from standard chemotherapeutic agents.^23^, ^25^, ^26^

### 4.1 Study limitations

Due to the nature of refractory mPDAC, patient participation in clinical trials is often short, which poses many study challenges. Our patient sample size was typical of a Phase II second or third‐line study in patients with mPDAC. However, our study was particularly heterogeneous in its line of therapy; patients were undergoing treatment with SM‐88 Regimen following anything from 1 to 7 prior lines of treatment. Although we report compelling trends indicating differences in response and OS between lines of SM‐88 Regimen, our study was not powered to define these differences statistically.

## 5 CONCLUSION

SM‐88 Regimen was well tolerated and had encouraging effects on QOL, especially considering many patients were near the end of life. The QOL effect was more pronounced among patients given the 920 mg/day dose. Taken together, preliminary disease control, survival, safety, and QOL results support exploring SM‐88 Regimen in the second line at a 920 mg/day dose for patients with PDAC, alone and possibly in combination with other agents.

## CONFLICT OF INTEREST

Dr. Marcus Smith Noel declares that he has held a Consulting or Advisory Role at Celgene, Taiho Pharmaceutical, and Ipsen. He is also a member of the Speakers Bureau for Celgene, Taiho Pharmaceutical, and Daiichi Sankyo/Astra Zeneca.

Semmie Kim declares Employment, Stock, and other Ownership Interests from TYME Inc.

Marion L. Hartley, PhD declares no potential competing interests.

Dr. Steve Wong declares no potential competing interests.

Dr. Vincent Picozzi declares that he has held a Consulting or Advisory Role at TriSalus Life Sciences. He also has Stock and other Ownership Interests in Amgen, Johnson and Johnson, McKesson, Thermo Fisher Scientific, and Cigna. He has received Research Funding from FibroGen, Celgene, Ipsen, NovoCure, Merk, TYME Inc., Abbvie, and NGM Biopharmaceuticals.

Dr. Harry Staszewski declare no potential competing interests.

Dr. Dae Won Kim has received Research Funding from Bold Therapeutics.

Dr. Jan Van Tornout declares employment at TYME Inc. Stock and other Ownership Interests from Bristol‐Myers Squibb, Novartis, Sanofi, TYME Inc., Lilly, and Merck. Has received an Honoraria from TYME Inc. Has held a Consulting or Advisory Role at TYME Inc.

Dr. Philip Agop Philip has held a Consulting or Advisory Role at Celgene, Ipsen, Merck, TriSalus, Daiichi, Syncore, and Taiho. He has been on the Speakers' Bureau for Celgene, Bayer, Ipsen, Novartis, and Incyte. Has received reimbursement for travel, accommodation, and expenses from Rafael Pharmaceuticals, Celgene, and Abbvie. He has received an Honoraria from Celgene, Bayer, Ipsen, Merck, AstraZeneca, TriSalus Life Sciences, Blueprint Medicines, Syncore, and Array BioPharma. He has received Research Funding from Bayer, Incyte, Karyopharm Therapeutics, Merck, Taiho Pharmaceutical, Momenta Pharmaceuticals, Novartis, Plexxikon, Immunomedics, Regeneron, Genentech, TYME Inc., Caris Life Sciences, ASLAN Pharmaceuticals, QED Therapeutics, Halozyme, Boston Biomedical, Advanced Accelerator Applications, Lilly, Taiho Pharmaceuticals, and Merus.

Dr. Vincent Chung has held a Consulting or Advisory Role at Ispen, Gristone Oncology, Westwood Bioscience, and Apeiron Biologics. He has been on the Speakers' Bureau for Ipsen and Celgene. He has received Research Funding from Roche and Merck.

Dr. Allyson J. Ocean has held a Consulting or Advisory Role at TYME Inc., and Celgene. She has been on the Speakers' Bureau at Daiichi Sankyo. Has received reimbursement for travel, accommodation, and expenses from Daiichi Sankyo.

Dr. Andrea Wang‐Gillam declares employment at Jocobio. She has held a Consulting or Advisory Role at AstraZeneca, Merck, Bayer, and Inxmed. Stock and other Ownership Interests at Jacobio. She has received Research Funding from AstraZeneca, Pfizer, Lilly, Verastem, Merck, BioMed Valley Discoveries, Roche, Bristol‐Myers Squibb, Xcovery, Boston Biomedical, Hutchinson MediPharma, Rafael Pharmaceuticals, Gossamer Bio.

## AUTHOR CONTRIBUTIONS

Steve Wong, Vincent J. Picozzi, Harry Staszewski, Dae Won Kim, Philip Agop Philip, Vincent Chung, Allyson J. Ocean, Andrea Wang‐Gillam, and Marcus S. Noel were principal site investigators in this trial and contributed to the writing of the manuscript. Marion L. Hartley, Jan M. Van Tornout, Semmie Kim, and Marcus S. Noel contributed to the analysis of data, interpretation of the findings, and the writing of the manuscript.

## ETHICS APPROVAL AND CONSENT TO PARTICIPATE

This study received approval from institutional IRBs, and all subjects provided written informed consent to participate. In addition, the study was performed in accordance with the Declaration of Helsinki.

## CONSENT FOR PUBLICATION

N/A.

## Data Availability

Upon reasonable request and subject to certain conditions, TYME Technologies, Inc. may provide access to de‐identified data from sponsored clinical trials that support the findings of this study.
